# The Clinical Values of Insulin-Like Growth Factor-1 and Insulin-Like Growth Factor Binding Protein-3 Levels in Blood and Thyroid Nodules

**DOI:** 10.1155/2017/3145234

**Published:** 2017-09-07

**Authors:** Ayfer Altas, Fatih Kuzu, Dilek Arpaci, Mustafa Unal, Murat Can, Figen Barut, Furuzan Kokturk, Sevil Uygun Ilikhan, Taner Bayraktaroglu

**Affiliations:** ^1^Department of Internal Medicine, Faculty of Medicine, Bulent Ecevit University, Zonguldak, Turkey; ^2^Division of Endocrinology and Metabolism, Department of Internal Medicine, Faculty of Medicine, Bulent Ecevit University, Zonguldak, Turkey; ^3^Department of Biochemistry, Faculty of Medicine, Bulent Ecevit University, Zonguldak, Turkey; ^4^Department of Pathology, Faculty of Medicine, Bulent Ecevit University, Zonguldak, Turkey; ^5^Department of Biostatistics, Faculty of Medicine, Bulent Ecevit University, Zonguldak, Turkey

## Abstract

**Aim:**

Insulin-like growth factor-1 (IGF-1) is a potent mitogen for many cells. IGF-1 plays a role in the pathogenesis of various tumors with its mutagenic and antiapoptotic properties. The aim of this study was to determine both the serum and intranodular levels of IGF-1 and insulin-like growth factor binding protein-3 (IGFBP-3) in patients with nodular thyroid diseases.

**Materials and Methods:**

In this study, 80 subjects who performed fine-needle aspiration biopsy (FNAB) were required in order to investigate the effects of serum and intranodular IGF-1 and IGFBP-3 in the pathogenesis of nodules. After performing FNAB, IGF-1 and IGFBP-3 levels were determined in blood and aspiration samples.

**Results:**

The serum levels of IGF-1 (232.8 ± 12.9 ng/ml) and IGFBP-3 (4.8 *μ*g/ml) were found significantly higher than that of the intranodular IGF-1 (39.1 ng/ml) and intranodular IGFBP-3 levels (0.173 *μ*g/ml) (*p* < 0.01). Intranodular levels of IGF-1 and IGFBP-3 were higher in subjects with multinodular thyroid gland than those of subjects with solitary nodules (*p* = 0.043). A positive correlation between the nodule size and the serum IGFBP-3 levels was detected (*p* = 0.042, *r* = 0.23).

**Conclusion:**

This study demonstrated the possible role of both IGF-1 and IGFBP-3 in the growth and the formation of multinodularity of thyroid nodules.

## 1. Introduction

A thyroid nodule is a common enhancing endocrinological condition. A thyroid nodule occurs through the excessive proliferation of epithelial hyperplasia and follicle cells [1]. Currently, the molecular mechanism induces the growth of some follicular cells; the reason for its localization in nodular goiter rather than normally functioning thyroid gland remains unclear. The most accepted hypothesis suggests the enlargement of the thyroid is a result of long-term stimulation of the thyroid-stimulating hormone (TSH) [2]. Increasing of TSH due to iodine deficiency, growth-stimulating immunoglobulins, and some other growth-stimulating factors stimulates thyroid enlargement [2].

Insulin-like growth factors (IGF-1) are important hormones that were first discovered in 1978 and have various actions in different tissues and organs. Most of the circulating IGF-1 are produced in the liver and transported to other tissues where they act as endocrine hormones [3]. IGF-1 is tightly bound to its own IGF-binding plasma proteins both in circulation and also in tissues. This prolongs the half-life of IGF-1 in circulation. All these binding proteins are available in plasma. IGF-binding protein-3 (IGFBP-3) is responsible for the binding of 95% of IGF-1 in circulation [4–7].

IGF-1 is a potent mitogen for many cells. It can promote cell proliferation, differentiation, and apoptosis as well as transformation, infiltration, and metastasis of tumor cells [8].

Insulin-like growth factor-1 (IGF-1) has been implicated in tumor cell apoptosis, transformation, invasion, and metastasis; however, its role in thyroid nodules is undetermined.

Many recent studies have reported an increase in IGF-1 production both in benign and malignant thyroid tumors [9, 10]. IGF-1 plays a role in the pathogenesis of various tumors with its mutagenic and antiapoptotic properties [11]. Exposure to high IGF-1 levels is likely to play a role in the acromegalic patients [12]. In vivo studies investigating IGF-1 in thyroid neoplasms are limited [13].

This prospective study aimed to determine the levels of IGF-1 and IGFBP-3 both intranodular and in the serum of patients with nodules in the thyroid gland who were planned to undergo biopsy, to investigate the relationship between expression of IGF-1 and thyroid nodule, and to evaluate the role of IGF-1 in differential diagnosis and pathogenetic function of benign and malignant thyroid nodules.

## 2. Materials and Method

### 2.1. Case Selection

This prospective study was performed in Bulent Ecevit University Health Application and Research Center, Department of Endocrinology and Metabolism between March 2012 and May 2013. It included 80 patients aged between 26 and 82 years of age who were admitted to our hospital for nodular goiter. This study was approved by the local Institutional Review Board (Date: 20/03/2012, Number: 2012/06), and written informed consent was obtained from every patient included in this trial.

Height and weight of the patients were measured and their body mass indexes were calculated.

The subjects underwent measurement for fasting TSH, free triiodothyronine (FT3), and free thyroxine (FT4) from a morning blood sample drawn from the antecubital vein. TSH, FT3, and FT4 levels were measured via the chemiluminescence method with Immulite 2000 device (Siemens Diagnostic, Los Angeles, CA, USA) at the Department of Biochemistry. The normal levels for FT3, FT4, and TSH were accepted as 1.8–4.7 pg/ml, 0.8–2.6 pg/ml, and 0.4–4 mIU/ml, respectively.

Thyroid ultrasonography tests were performed with a high-resolution ultrasonography device with a 7.5 MHz probe. Sonographic findings of the thyroid nodules were grouped as benign and suspected malignancy. Definition of suspected malignancy included thyroid nodules with a size of greater than 4 cm, hypoechoic texture, a thick hypoechoic halo, microcalcification, prominent peripheral vascularization, and irregular borders. The sonographic findings and laboratory hormone tests of the nodules were compared.

Two groups designated as hypoactive nodule and hyperactive nodule were formed according to nodule characteristics on thyroid scintigraphy.

Verbal and written informed consent were obtained from each patient. The fine-needle aspiration biopsy (FNAB) procedures were carried out at the Department of Endocrinology and Metabolism. Specimen of thyroid washout was diluted with 1 cc saline. The pathological preparations obtained during biopsy were fixed with alcohol. They were examined at the pathology laboratories of our hospital.

Venous blood samples were drawn and centrifuged at 5000 rpm for 5 minutes for IGF-1 and IGFBP-3 measurements in the study group.

Thyroid FNAB materials were centrifuged at 2000 rpm for 5 minutes and stored in a deep freezer at −70 degrees. After collecting all serum and aspiration materials, serum and aspirate IGF-1 levels were studied using commercially available OmniKine Human IGF-1 ELISA kit (Assay Biotechnology Company, CA, USA).

The OmniKine™ Murine IGF-1 ELISA kit was tested with IGF-BP1, IGF-BP2, IGF-BP3, IGF-BP4, IGF-BP5, IGF-BP7, and IGF-2 and exhibited less than 1% cross reactivity.

The test results were calculated with the calibration curve formed by 31.25, 62.5, 125, 250, 500, 1000, and 2000 pg/ml of standards. The study was performed with the BioTek-branded (Vermont, USA) ELx50 washing device, and the ELISA plaques were read with an ELx800 ELISA plaque reader device at 450 nm. The measurement range of the test was 31.25 to 2000 pg/ml and its analytic sensitivity was 32 pg/ml. Serum and aspirate IGFBP-3 levels were studied using commercially available Human IGFBP-3 ELISA kit (Boster Biological Technology, CA, USA). The test results were calculated according to the calibration curve drawn with standards with a concentration of 0.0, 156.2, 312.5, 625, 1250, 2500, 5000, and 10,000 pg/ml. The study was performed with the BioTek-branded (Vermont, USA) ELx 50 washing device. ELISA plaques were read at 450 nm with an ELx800 ELISA plaque reader device. The measurement range of the test was 0 to 10,000 pg/ml.

Cytopathology results of thyroid FNAB specimens were classified benign (Bethesda II) or with suspicious malignancy (atypia of unknown significance or follicular lesion of unknown significance—Bethesda III, suspicious for follicular neoplasm—Bethesda IV, malignancy suspicious—Bethesda V, and malignant cytology—Bethesda VI) [14].

Inclusion criteria were as follows: subjects aged over 18 years who were admitted to the endocrinology clinic with >1 cm thyroid nodules were included.

Exclusion criteria were as follows: diseases that resulted from the excess or deficiency of growth hormones such as acromegaly or growth hormone (GH) deficiency were excluded in this study. Also, patients with known any malignancy were excluded from the study. Pregnant and breastfeeding women were also excluded.

### 2.2. Statistical Analysis

Statistical analyses were performed with SPSS 18.0 (SPSS Inc., Chicago, IL, USA) software package. Shapiro-Wilk test was used to check whether numerical variables were normally distributed. The descriptive statistics included mean ± standard deviation and median (minimum-maximum) for numerical variables and number and percentage for categorical data. Comparison of two groups with respect to numerical variables was carried out using Student's *t*-test when parametric test assumptions were met or Mann–Whitney *U* test when those assumptions were not met. Comparison of three or more groups with regard to numerical variables was performed with one-sided analysis of variance when the parametric test assumptions were met or Kruskal-Wallis analysis of variance test when those assumptions were not met. Paired comparison of the groups was performed with Tukey's test when the groups significantly differed in one-sided analysis of variance. Paired comparisons of the subgroups in Kruskal-Wallis analysis of variance were carried out with Mann–Whitney *U* test with Bonferroni correction. The linear relationship between two numerical variables was performed with Pearson correlation analysis when the parametric test assumptions were met or Spearman's correlation analysis when those test assumptions were not met. The results were analyzed in a confidence interval of 95% and a *p* value less than 0.05 was considered statistically significant.

## 3. Results

This study included 60 females (75%) and 20 males (25%). The mean age of the patients was 55.2 ± 13.3 years. BMI was 29.05 ± 4.57 kg/m^2^; TSH was 0.9 mIU/ml (0.4–3.3 mIU/ml); FT4 was 1.01 ng/dl (0.01-2.6 ng/dl), FT3 was 3.5 pg/ml (1.5–7.7 pg/ml); serum IGF-1 was 232.8 ± 12.9 ng/ml; serum IGFBP-3 was 4.8 *μ*g/ml (2.2–6.4 *μ*g/ml); intranodular IGF-1 was 39.1 ng/ml (32.6–49.8 ng/ml), and intranodular IGFBP-3 was 0.173 *μ*g/ml (0.07–0.64 *μ*g/ml).

There was a particularly strong and positive correlation between serum IGF-1 and intranodular IGF-1 levels (*p* = 0.000, *r* = 0.42, Table 1).

Although there were no differences between age, BMI, thyroid status, serum TSH, FT3, and FT4 in both sexes, mean serum IGF-1 in males was significantly lower when compared to that of the females (*p* = 0.028). However, there was no significant difference between both sexes with respect to serum IGFBP-3, intranodular IGF-1, and intranodular IGFBP-3 (*p* = 0.768, *p* = 0.969, and *p* = 0.876) (Table 2).

No significant difference was evident in the comparison of serum and intranodular IGF-1 and IGFBP-3 levels based on age (Table 2). However, the groups of the study subjects based on the results of thyroid function tests revealed that only the subjects with hypothyroidism had a positive correlation between age and serum IGF-1 level (*r* = 0.67, *p* = 0.023). A strong positive correlation was observed between age and intranodular IGF-1 level (*r* = 0.77, *p* = 0.006) and serum IGF-1 levels (*r* = 0.67, *p* = 0.023) in the hypothyroidism group.

There existed no significant correlation between BMI and the levels of IGF-1 and IGFBP-3 (*p* > 0.05) in both the serum and intranodular samples.

Serum IGF-1 levels (232.8 ± 12.9) were significantly higher than intranodular IGF-1 levels [39.1 (32.6–49.8)] in the 80 subjects included in the study (*p* = 0.001). Similarly, serum IGFBP-3 level [4.8 (2.2–6.4)] was significantly higher than intranodular level [0.173 (0.07–0.64)] (*p* = 0.001).

Out of 56.3% (*n* = 45) of patients have euthyroidism, 30% of patients (*n* = 24) have hyperthyroidism, and 13.7% of patients (*n* = 11) have hypothyroidism. There was no significant difference between hyperthyroid and hypothyroid subjects with respect to serum and intranodular IGF-1 and IGFBP-3 levels (for serum IGF-1 level, *p* = 0.265; for intranodular IGF-1, *p* = 0.351; for serum IGFBP-3, *p* = 0.215; and for intranodular IGFBP-3, *p* = 0.820).

Intranodular IGF-1 and IGFBP-3 levels were significantly higher in the subjects with multinodular goiter compared to single nodules (*p* = 0.001 and *p* = 0.043, resp.) (Table 2). However; serum IGF-1 and IGFBP-3 levels were similar in both groups (*p* = 0.191 and *p* = 0.841, resp.). There were no significant differences between the subjects with suspicious findings and the subjects with benign findings on thyroid ultrasonography with respect to serum and intranodular IGF-1 and IGFBP-3 levels (*p* > 0.05).

Cytopathology results of 73 cytology specimens (81.2%) were benign (Bethesda II), 4 (5%) were atypia of unknown significance or follicular lesion of unknown significance (Bethesda III), 2 (2.5%) were suspicious for follicular neoplasm (Bethesda IV), and 1 (1.25%) was differentiated thyroid cancer (Bethesda VI). No significant differences existed between the subjects with benign aspiration cytology and the subjects with suspicious or malignant aspiration cytology with respect to serum IGF-1, IGFBP-3, and intranodular IGF-1 and IGFBP-3 levels (*p* > 0.05).

Of the subjects included in the study, 15 (16.2%) were operated with thyroid surgery (subtotal or total thyroidectomy). According to the pathology results, 2 (2.5%) cases had follicular adenoma, 9 (10.0%) had nodular hyperplasia, and 4 (3.75%) had differentiated thyroid cancer. There were no differences between benign/malign histopathologic results and IGF-1/IGFBP3 in serum (*p* = 0.983 and *p* = 0.436, resp.); in addition, there were no differences of IGF-1/IGFBP-3 in nodules (*p* = 0.966 and *p* = 0.477, resp.). No significant correlation was detected between the sizes of thyroid nodules and serum IGF-1 (*r* = −0.032, *p* = 0.771), between the sizes of IGF-1 in nodules (*r* = −0137, *p* = 0.225), and also between the sizes of thyroid nodules and IGFBP3 in nodules (*r* = −0075, *p* = 0.509). There was a weak positive correlation in the correlation analysis between the nodule size and serum IGFBP-3 levels (*p* = 0.042, *r* = 0.23, Table 1).

When we identified the cutoff for IGFBP-3 in biopsy specimen, we detected (0.111) that the area under the curve (AUC) was 0.590; *p* = 0.391. The sensitivity was 42.86%; specificity was observed as like 84.9%. In addition, we detected for IGF-1 in biopsy specimen, we observed 41.8; AUC was 0.636, *p* = 0.178; specificity was 100%; and specificity was 32.8%. We compared the values to determine the upper and lower cutoff value of cytology and found that it was not significantly different.

The correlation analyses of the subjects' age, BMI, nodule size, and serum and intranodular IGF-1 and IGFBP-3 levels were shown (Table 1).

## 4. Discussion

Our study demonstrated that the intranodular IGF-1 and IGFBP-3 levels were significantly higher in the subjects with multinodular goiter compared to single nodules. Also, we found that size of thyroid nodules and serum IGF1BP3 levels were positively correlated. But we did not find any relationship between the results of histopathology and serum/intranodular IGF-1/IGFBP3 levels.

Our study found that intranodular IGF-1 and IGFBP-3 levels were significantly higher in subjects with multinodular thyroid gland compared to subjects with solitary nodules which may indicate the role of IGF-1 and IGFBP-3 in nodular goiter pathogenesis. Studies on patients with acromegaly also demonstrated an enhancement in IGF-1 levels and the rate of multinodular goiter, suggesting a goitrogenic action of IGF-1 [15].

IGF-1 has an important function in development and functioning of normal thyroid follicular cells in vitro [16]. IGF-1 stimulates protein and DNA synthesis of thyrocytes, fibroblasts, follicular cells, and endothelial cells in the thyroid gland [17]. Particularly, IGF-1 has an active role in the proliferation of TSH-induced thyrocyte proliferation via autocrine mechanism and a synergistic effect with TSH [18–20]. Also, IGF-1 plays an important role in thyrocyte proliferation, differentiation, and malignant transformation [21]. In vitro studies both in animals and human beings have shown the mitogenic effects of IGF-1 on thyrocytes [22]. In a study with mice thyroid cell cultures, Tramontano et al. [23] demonstrated that the in situ expression level of IGF-1 was lower in normal thyroid tissue than the cancerous thyroid tissue. In addition to this, they reported that TSH inhibits IGFBP-3 secretion. Inhibitors of thyroid gland function usually increase the synthesis of IGFBP-3 [17]. Although there are opposing views regarding the role of IGF-1 and IGFBP-3 in carcinogenesis, a series of epidemiological studies have linked high levels of circulating IGF-1 level and altered IGFBP-3 level to increased malignancy potential in many organs (breast, prostate, lung, colon, and rectum) [16, 24]. Age, nutritional status, and genetic and metabolic factors affect levels of circulating IGF-1 and IGFBP-3. Xu et al. [25] found evidence of a possible epidemiological role of polymorphic IGFBP-3 in the development of differentiated thyroid cancer.

In acromegaly patients, serum IGF-1 levels are high and they tend to have more multinodular diseases than other subjects. This explains the role of IGF-1 in the pathogenesis of nodule formation [15, 26]. In addition, to explain the effects of IGF-1 in the thyroid tissue, the prevalence of goiter in pygmies who has low IGF-1 levels is low, although the lack of advanced iodine is apparent [27].

In our study, serum IGF-1 levels were found to be significantly higher than intranodular IGF-1 levels. Similarly, serum IGFBP-3 levels were also significantly higher than intranodular IGFBP-3 levels. Previous studies also reported the effect of gender differences for both the GH and IGF-1 levels. In recombinant GH-administered patients, it was found that the male thyroid tissue due to lean body mass and insulin sensitivity was more sensitive to IGF-1 effects than its female counterpart [28]. Volzke et al. [29] reported significantly higher serum IGF-1 levels in men compared to women with nodular thyroid disease. In contrast to their results, in our study, females had significantly higher serum IGF-1 levels compared to males. This might be due to the hormonal alterations. In another study performed by Liu et al., no differences for IGF-1 levels related to gender differences were found. In addition to this, there were no significant differences between both genders with respect to age and body mass index similar to previous results [30]. Similar to these results, we did not find any relationship between serum IGF-1/IGFBP3 levels and age/BMI.

Minuto et al. [10] measured IGF-1 levels with radioactive binding method in surgical thyroid specimens. They found that IGF-1 levels were higher in modular and cancerous thyroid tissues than normal thyroid tissue samples. Based on these results, the authors suggested a possible role of IGF-1 in the pathogenesis of nodular goiter. Another study performed by Khandwala et al. [22] showed autocrine IGF-1 production in papillary thyroid cancer cells and also demonstrated IGF-1 immunoreactivity in thyroid cell clone medium. Yashiro et al. [31] detected IGF-1 receptors in malignant thyroid epithelium as well as in normal thyroid epithelium. IGF-1 binding was greater in cancer specimens compared to benign and normal thyroid specimens in thyroid surgical preparations. Khandwala et al. [22] demonstrated the mitogenic effects of IGF-1 in human papillary thyroid cancer cells. IGF-1 led to a 200% increase in growth. In contrast to these studies, we did not find any significant difference between serum and intranodular IGF-1 and IGFBP-3 levels of subjects with suspicious malignancy and benign features by ultrasonographic evaluation; also we did not observed any differences between serum and intranodular IGF-1 and IGFBP-3 levels in subjects with benign and suspicious/malignant fine needs aspiration cytology. The limitation of our study was the absence of normal subjects.

Serum IGF-1 levels decrease with aging. The risk of goiter has been found to be increased with aging. According to our results, no statistically significant difference was found in serum IGF-1 levels between subjects aged over or lower than 45 years of age. This may be due to the unequal numbers of subjects (*n* = 17 versus *n* = 63). When the subjects who were diagnosed with hypothyroidism were evaluated as a separate group, a positive correlation was found between the age and the level of serum IGF-1. Similarly, there was a positive correlation between age and intranodular IGF-1 levels. It was considered that a rise of IGF-1 level took place with aging in patients with hypothyroidism. According to our best knowledge, there is no data suggesting a correlation between age and serum IGF-1 levels in hypothyroidism and nodular thyroid disease.

Iglesias et al. [16] reported a strong correlation between low levels of IGF-1 and IGFBP-3 and hypothyroidism. They concluded that the thyroid function affects GH-IGF axis. We did not find any significant relation between IGF-1 and IGFBP-3 levels in hypothyroid and hyperthyroid patients. We suggest that the reason of the lack of such correlation was the euthyroid status in most of the patients and the low number of subjects with hyperthyroidism and hypothyroidism.

Our study did not demonstrate any significant relationship between the scintigraphic findings (hypoactive-hyperactive) and serum and intranodular IGF-1 level. Similar to our results, Eszlinger et al. [8] examined the expression of growth factors in hot and cold thyroid nodules and reported no significant difference between the IGF-1 levels in hypoactive and hyperactive nodules compared to the surrounding tissues. Similarly, another study found no significant relationship between the scintigraphic findings of thyroid nodules and positivity and the level of IGF-1 uptake in thyroid nodules [32]. These findings suggest that there is no relationship between IGF-1 and scintigraphic findings.

There was a significant positive correlation between nodule size and serum IGFBP-3 levels in our study. While there was no significant correlation between IGF-1 levels and nodule size, the results demonstrated a mild increase in IGFBP-3 levels in parallel to nodule size enhancement. The literature results about the relation between IGF-1 and thyroid nodule size were controversial. Some studies suggested that there was also no significant correlation between thyroid/nodules volumes and serum levels of IGF-1 similar to our results [15, 32]. In a pediatric study, IGF-1 levels in children with normal thyroid volume versus increased thyroid volume were compared. Children with goiter were found to have higher levels of IGF-1 [33]. Another study found a positive correlation between tumor size and IGF-1 involvement in thyroid cancers [20].

## 5. Conclusion

In our study, we found no correlation between serum and intranodular IGF-1 and IGFBP-3 levels and thyroid cytology results/thyroid status/scintigraphic feature of nodules. Intranodular IGF-1 and IGFBP-3 levels were significantly higher in subjects with multinodular goiter compared to the ones with single nodules. There was a positive correlation between nodule size and serum IGFBP-3 level. According to our results, we suggested that a higher IGF-1 level in multinodular cases as well as an increase in IGFBP-3 level that was parallel to an increase in thyroid nodule size were the findings suggesting the role of IGF-1 and IGFBP-3 in the pathogenesis of nodular goiter. It needs more studies with more subjects to evaluate the effects of IGF-1/IGFBP-3 levels on thyroid nodules and the risk of thyroid malignancy.

## Figures and Tables

**Table 1:** Correlation analyses of age, BMI, nodule size, and serum and intranodular IGF-1 and IGFBP-3 results.

Parameters	Serum IGF-1 (*r*/*p*)	Intranodular IGF-1 (*r*/*p*)	Serum IGFBP-3 (*r*/*p*)	Intranodular IGFBP-3 (*r*/*p*)
Age	−0.07/0.955^∗∗^	0.01/0.921^∗^	−0.04/0.718^∗^	−0.06/0.614^∗^
BMI	0.2/0.868^∗∗^	−0.01/0.962^∗^	0.19/0.388^∗^	−0.01/0,928^∗^
Nodule size	0.03/0.777^∗^	−0.14/0.225^∗^	**0.23/0.042** ^∗^	−0.08/0.509^∗^
Serum IGF-1		**0.42/0.000** ^∗^	−0.11/0.343^∗^	−0.06/0.628^∗^
Intranodular IGF-1			−0.09/0.417^∗^	−0.09/0.397^∗^
Serum IGFBP-3				0.12/0.283^∗^

^∗∗^Pearson correlation analysis. ^∗^Spearman's correlation analysis.

**Table 2:** Demographic, ultrasonographic, and cytological results thyroid nodules; comparison of serum IGF-1; intranodular IGF-1, serum IGFBP-3, and intranodular IGFBP-3.

Clinic and laboratory features	Serum IGF-1 (ng/ml)	Intranodular IGF-1 (ng/ml)	Serum IGFBP-3 (*μ*g/ml)	Intranodular IGFBP-3 (*μ*g/ml)
<45 years (*n* = 17)	234.2 ± 10.6	37.7 (34.4–47.6)	4.8 (2.2–6.3)	0.173 (0.08–0.48)
>45 years (*n* = 63)	232.5 ± 13.6	39.1 (32.6–49.8)	4.9 (2.4–6.4)	0.173 (0.06–0.64)
*p*	0.638^∗∗^	0.823^∗∗∗^	0.680^∗∗∗^	0.828^∗∗∗^
Female	234.6 **±** 13.2	38.8 (33.7–49.8)	4.85 (2.2–6.3)	0.208 (0.07–0.64)
Male	227.5 **±** 10.6	39.1 (32.6–48.7)	4.8 (3.8–6.4)	0.172 (0.07–0.50)
*p*	**0.028** ^∗∗^	0.969^∗∗∗^	0.768^∗∗∗^	0.876^∗∗∗^
Single [*n* = 20 (25%)]	229.5 ± 12.9	37.7 ± 2.6	4.8 (3.5–6.3)	0.14 (0.07–0.39)
Multinodular [*n* = 60 (75%)]	233.9 ± 12.9	40.7 ± 4.1	4.8 (2.2–6.4)	0.19 (0.07–0.64)
*p*	0.191^∗∗^	**0.001** ^∗∗^	0.841^∗∗∗^	**0.043** ^∗∗∗^
Benign sonography	236.1 **±** 13.7	40.05 **±** 4.82	4.6 (2.4–6.3)	0.175 (0.07–0.5)
Sonography revealing suspected malignancy	231.5 **±** 12.5	39.9 **±** 3.7	4.9 (2.2–6.4)	0.173 (0.07–0.64)
*p*	0.145^∗∗^	0.899^∗∗^	0.577^∗∗∗^	0.674^∗∗∗^
Hypoactive [*n* = 18 (22.5%)]	234.5 (212–278)	39.9 ± 3.6	5.05 (2.4–6.3)	0.17 (0.07–0.5)
Hyperactive [*n* = 44 (55%)]	230.5 (208–254)	40.2 ± 4.9	5.2 (2.2–6.4)	0.14 (0.07–0.64)
*p*	0.545^∗∗∗^	0.773^∗∗^	0.895^∗∗∗^	0.187^∗∗∗^
Benign cytology (*n* = 73, 91.3%)	232.5 **±** 12.9	39.1 (33.7–49.8)	4.8 (2.2–6.4)	0.173 (0.07–0.64)
Suspicious and malignant cytology (*n* = 7, 8.7%)	236.3 **±** 13.9	37.9 (32.6–41.9)	5.4 (4.1–6)	0.173 (0.08–0.29)
*p*	0.247	0.237	0.528	0.433
Histopathology				
Benign (*n* = 11)	234 ± 11.3	40.7 ± 5.01		0.23 ± 0.15
Malign (*n* = 4)	234.5 ± 7.0	40.8 ± 2.29		0.169 ± 0.09
*p*	0.983^∗∗^	0.966^∗∗^	0.436^∗∗^	0.477^∗∗^

^∗∗∗^Mann–Whitney *U* test. ^∗∗^Student's *t*-test.

## References

[1] Alpay H. C., Kalidag T., Keles E., Kaygusuz I., Yalcin S., Kapusuz Z. (2007). The effects of fine-needle biopsy on thyroid hormone levels. *Otolaryngology and Head and Neck Surgery*.

[2] Tezelman S. T., Siperstein A. E., Clark O. H., Duh Q. Y. (1997). Signal transduction in thyroid neoplasms. *Textbook of Endocrine Surgery*.

[3] Wang Y., Nishida S., Sakata T. (2006). Insulin-like growth factor-I is essential for embryonic bone development. *Endocrinology*.

[4] Baker J., Liu J. P., Robertson E. J., Efstratiadis A. (1993). Role of insulin-like growth factors in embryonic and postnatal growth. *Cell*.

[5] Torres-Aleman I. (1999). Insulin-like growth factors as mediators of functional plasticity in the adult brain. *Hormone and Metabolic Research*.

[6] Turgut G., Kaptanoğlu B., Turgut S., Genc O., Tekintürk S. (2003). Influence of acute exercise on urinary protein, creatinine, insulin-like growth factor-I (IGF-I) and IGF binding protein-3 concentrations in children. *The Tohoku Journal of Experimental Medicine*.

[7] Turgut S., Kaptanoğlu B., Emmungil G., Turgut G. (2006). Increased plasma levels of growth hormone, insulin-like growth factor (IGF)-I and IGF-binding protein 3 in pregnant rats with exercise. *The Tohoku Journal of Experimental Medicine*.

[8] Eszlinger M., Krohn K., Kratzsch J., Voigt C., Paschke R. (2001). Growth factor expression in cold and hot thyroid nodules. *Thyroid*.

[9] Fujimoto J., Brenner-Gati L. (1992). Protein kinase-C activation during thyrotropin-stimulated proliferation of rat FRTL-5 thyroid cells. *Endocrinology*.

[10] Minuto F., Barreca A., Del Monte P., Cariola G., Torre G. C., Giordano G. (1989). Immunoreactive insulin-like growth factor 1 (IGF-1) and IGF-1-binding protein content in human thyroid tissue. *The Journal of Clinical Endocrinology and Metabolism*.

[11] Decuypere E., Van Asa P., Van der Geyten S., Darras V. M. (2005). Thyroid hormone availability and activity in avian species: a review. *Domestic Animal Endocrinology*.

[12] Tita P., Ambrosio M. R., Scollo C. (2005). High prevalence of differentiated thyroid carcinoma in acromegaly. *Clinical Endocrinology*.

[13] Wynford-Thomas D. (1993). Molecular basis of epithelial tumorigenesis: the thyroid model. *Critical Reviews in Oncogenesis*.

[14] Cibas E. S., Ali S. Z., NCI Thyroid FNA State of the Science Conference (2009). The Bethesda system for reporting thyroid cytopathology. *American Journal of Clinical Pathology*.

[15] Cannavò S., Squadrito S., Finocchiaro M. D. (2000). Goiter and impairment of thyroid function in acromegalic patients: basal evaluation and follow-up. *Hormone and Metabolic Research*.

[16] Iglesias P., Bayon C., Mendez J., Gancedo P. G., Grande C., Diez J. J. (2001). Serum insulin-like growth factor type 1, insulin-like growth factor-binding protein-1, and insulin-like growth factor-binding protein-3 concentrations in patients with thyroid dysfunction. *Thyroid*.

[17] Vesely D., Astl J., Latuvka P., Matucha P., Sterzl I., Betka J. (2004). Serum levels of IGF-I, HGF, TGFbeta1, bFGF and VEGF in thyroid gland tumors. *Physiological Research*.

[18] Burikhanov R., Coulonval K., Pirson I., Lamy F., Dumont J. E., Roger P. P. (1996). Thyrotropin via cyclic AMP induces insulin receptor expression and insulin co-stimulation of growth and amplifies insulin and insulin-like growth factor signaling pathways in dog thyroid epithelial cells. *The Journal of Biological Chemistry*.

[19] Farid N. R., Shi Y., Zou M. (1994). Molecular basis of thyroid cancer. *Endocrine Reviews*.

[20] Maiorano E., Perlino E., Triggiani V., Nacchiero M., Giove E., Ciampolillo A. (1998). Insulin-like growth factor-1 and insulin-like growth factor receptor in thyroid tissues of patients with Graves’ disease. *International Journal of Molecular Medicine*.

[21] Vella V., Sciacca L., Pandini G. (2001). The IGF system in thyroid cancer: new concepts. *Molecular Pathology*.

[22] Khandwala H. M., McCutcheon I. E., Fylvbjerg A., Friend K. E. (2000). The effects of insulin-like growth factors on tumorigenesis and neoplastic growth. *Endocrine Reviews*.

[23] Tramontano D., Cushing G. W., Moses A. C., Ingbar S. H. (1986). Insulin-like growth factor-I stimulates the growth of rat thyroid cells in culture and synergizes the stimulation of DNA synthesis induced by TSH and Graves’-IgG. *Endocrinology*.

[24] Rodriguez-Arnao J., Miell J. P., Ross R. J. (1993). Influence of thyroid hormones on the GH-IGF-I axis. *Trends in Endocrinology and Metabolism*.

[25] Xu L., Mugartegui L., Li G. (2012). Functional polymorphisms in the insulin-like binding protein-3 gene may modulate susceptibility to differentiated thyroid carcinoma in Caucasian Americans. *Molecular Carcinogenesis*.

[26] Cheung N. W., Boyages S. C. (1997). The thyroid gland in acromegaly: an ultrasonographic study. *Clinical Endocrinology*.

[27] Peter H. J., Uburgi B., Gerber H., Ingbar S. H., Braverman L. E., Utiger R. D. (1996). Pathogenesis of non-toxic diffuse and nodular goiter. *Werner’s The Thyroid*.

[28] Giannoulis M. G., Boroujerdi M. A., Powrie J. (2005). Gender differences in growth hormone response to exercise before and after rhGH administration and the effect of rhGH on the hormone profile of fit normal adults. *Clinical Endocrinology (Oxford)*.

[29] Völzke H., Friedrich N., Schipf S. (2007). Association between serum insulin-like growth factor-I levels and thyroid disorders in a population-based study. *The Journal of Clinical Endocrinology and Metabolism*.

[30] Liu Y. J., Qiang W., Shi J., Liv S. Q., Ji M. J., Shi B. Y. (2013). Expression and significance of IGF-1 and IGF-1R in thyroid nodules. *Endocrine*.

[31] Yashiro T., Ohba Y., Murakami H. (1989). Expression of insulin-like growth factor receptors in primary human thyroid neoplasms. *Acta Endocrinologica*.

[32] Baştürk E., Kement M., Yavuzer D. (2012). The role of insulin-like growth factor 1 in the development of benign and malignant thyroid nodules. *Balkan Medical Journal*.

[33] Brzozowska M., Kinalska I., Kretowski A. (2005). The level of IGF-1 and TGF-beta-1 in the blood serum and the thyroid size in children with normal ioduria. *Endokrynologia, Diabetologia i Choroby Przemiany Materii Wieku Rozwojowego*.

